# Digital morphology analyzers in hematology: Comments on the ICSH review and recommendations

**DOI:** 10.1111/ijlh.13181

**Published:** 2020-03-09

**Authors:** Hans‐Inge Bengtsson

**Affiliations:** ^1^ Director Scientific Affairs CellaVision AB Lund Sweden

**Keywords:** digital imaging, laboratory hematology, laboratory standards, recommendations

Dear Editors,

We read the article “Digital morphology analyzers in hematology: ICSH review and recommendations” by Kratz et al^1^ published in this journal with great interest. While publication of a review article and recommendations for such a new and exciting technology was timely, there seems to have been some misunderstandings in the article which we would like to address. While the authors stated that in their article “strengths and weaknesses of digital imaging were determined,” our review revealed examples where the authors seemed to have misunderstood the findings of previous research. As a result, there is a potential that the reader might get a negative view of digital morphology in general and be discouraged from its use, which we believe was not the intention of this review. In order to highlight some of our concerns, we have selected a small number of examples to support our findings.

An example is the use of the word “detection” in the review article. The authors state “Briggs et al similarly reported poor detection of immature granulocytes, eosinophils, and basophils.”^1^ We found the use of the word “detection” misleading and wish to point out that Briggs et al^2^ did not use this word anywhere in their article. Furthermore, no statement is made on the detectability of these cell types, instead *correlation* between the manual and the automated differential, and preclassification accuracy is discussed. Briggs et al^2^ explained that lower *correlation* between the manual and the CellaVision^®^ DM96 (DM96) differential results for eosinophils was potentially due to “a staining issue which could be modified.” Regarding basophils and immature myeloid cells, they concluded that the lower correlation was due to the low number of counted cells (implying that the reason for the low correlation was partly in the manual method) as this part of the study was performed only counting 100 cells.^2^ In summary, Briggs et al concluded that “We have demonstrated that the differential from the DM96 is as good as that by a laboratory scientist; however, the laboratory scientist operating the DM96 must be skilled in blood cell morphology.”^2^

Another example where the use of the word “detection” by Kratz et al^1^ is misleading is the following statement: “In contrast, Park et al found that detection of basophils and band neutrophils was poor, but blast detection showed high sensitivity and specificity.” Regarding band neutrophils and basophils, Park et al^3^ actually refer to weaker correlation between manual microscopy and using the DM96 analyzer but judged this as satisfactory—not poor; they did not make any conclusions regarding the detection of these cell types. Park et al^3^ referred to the term “detection” when they did a concordance analysis for the following six abnormalities: “[ie, the presence of atypical lymphocytes, blasts, promyelocytes > 3%, myelocytes > 3%, and metamyelocytes > 3%, nucleated red blood cells (RBCs)].” The result according to Park et al was satisfactory, especially for blasts.^3^ The only time Park et al^3^ discuss “low detection” was in another context in relation to positive predictive value of promyelocytes. They also point out one case where two manual differential counts were completely discordant, while the suggestion of the DM96 was in agreement with one of the manual differential counts, which “was determined to be correct after review by the laboratory hematologist.”^3^

To conclude, the use of the word “detection” by Kratz et al^1^ in a context not consistent with the referenced articles may create incorrect perceptions about the capability of digital morphology analyzers.

We also found statements in the review article that were not supported by scientific references. An example is “The review speed may be expected to be slower in busy laboratories with large numbers of samples that have low WBC counts as well as abnormal cells,”^1^ in which case we did not manage to locate the source(s) of this statement in the scientific literature. In fact, the above article by Briggs et al^2^ clearly contradicts this statement showing that the average time for the analysis of 30 blood films—including samples with low WBC count and highly abnormal cells—was 1 hour 20 minutes using the DM96, while it was 2 hour 54 minutes by manual analysis. Time saving using the DM96 analyzer is also confirmed by other authors.^4^, ^5^ Similarly, the authors state without reference “Therefore, depending on the patient population, 10 to 20% of all slides may still require manual microscopic review.” It is unclear what these numbers are based on.

Some of the limitations mentioned in Table 2 of the review article (“Situations in which CellaVision has limitations and for which a manual microscopic review should be considered”)^1^ also seem to be in contradiction with references cited throughout the paper. For example, one of the limitations, “Suspicion of the presence of pathological cell types, including blasts, plasma cells, and immature granulocytes,”^1^ is not consistent with conclusions made by Briggs et al^2^ and Park et al,^3^ as discussed above. Similarly, another limitation in Table 2 of the review article, “All samples from newborns and from patients with leukemia,”^1^ seems to conflict with the findings of Herishanu et al.^6^ Furthermore, Marionneaux et al^7^ found that the DM96 can be used as “a feasible, rapid and inexpensive screening tool” to subclassify patients with CLL into typical or atypical CLL subgroups.

Furthermore, the recommendation “In the presence of abnormal cells or in pediatric samples of lymphocytes, the size, thickness, and color differences can lead to incorrect cell identification, often resulting in an overestimation of blast cells”^1^ refers to an article by Eilertsen et al^8^; however, our thorough review of this article did not find any statement or conclusion which supports this statement.

Regarding the review article's recommendations, we found it remarkable that while the authors mention that digital morphology analyzers display only a limited number of cells, they only recommend “to always reconcile the flags of the automated analyzer with the report of the digital imaging. In the presence of any discrepancy, a manual differential with the microscope should be performed”,^1^ instead of the obvious first choice of examining an increased number of cells as it is recommended by the CLSI document H20‐A2 standard.^9^ This would limit the number of slides requiring manual microscopy to those referred to in the authors' example, where pathological cells are located exclusively in the margins of the slide.

The review also sets out to propose recommendations for improvement of digital imaging; however, the standards they recommend for manufacturers to follow are mainly relevant for whole slide imaging (WSI) or other medical imaging technologies and not specific to digital morphology analyzers. While it was mentioned that these standards “are applicable to digital analyzers only in general terms and not as specific recommendations,”^1^ it would have been beneficial to point out specific parts of these standards the authors find applicable for digital morphology systems used in hematology. There is a considerable difference between WSI and other medical imaging technologies and digital morphology analysis in hematology which was not explained by the authors.

We also found that the authors of this review article might not have fully understood how digital morphology analyzers function today. At several places in the article, the authors seem to fault the analyzer for requiring a competent user for its operation and expect it to function as a fully automated self‐reporting device. As an example, the review criticizes the fact that the study written by Lee et al^10^ evaluated reclassified rather than preclassified results and concluded that “Therefore, the study may be unable to test the possibility of full automation of PBS [peripheral blood smear] analysis.”^1^ While Lee et al^10^ mentioned this as a limitation of their study, their stated aim was “to assess the ability of the CellaVision DM96 (DM96) system and software to classify leukocytes by comparing it with the manual PBS examination.” Just like manual microscopy, the DM96 system per design includes a competent user; therefore it is not correct to expect a fully automated digital morphology system as they are not currently designed to operate in that manner. Similarly, regarding the study by Criel et al,^11^ the authors of the review article comment “However, evaluation of the images by an experienced observer remained necessary,” suggesting that this was a negative property of the system.

Finally, the comment in the review article in relation to the two tables talks about “advantages of the CellaVision systems” and “Situations in which the CellaVision has limitations.”^1^ While we feel honored to be regarded as a synonym for digital morphology, it would be more appropriate to use “digital morphology” in the title of both Tables.^1^

There are a number of other comments we could have made regarding the review article; however, we omitted them from this letter. For those who are interested, we would be happy to share our complete list of comments.

## CONFLICT OF INTEREST

The author is an employee of CellaVision AB.
